# Optimizing Cross-Sectional Prediction of Social Functioning in Youth Referred for Neuropsychological Testing

**DOI:** 10.1371/journal.pone.0128303

**Published:** 2015-05-26

**Authors:** Matthew D. Lerner, Lauren M. Potthoff, Scott J. Hunter

**Affiliations:** 1 Department of Psychology, Stony Brook University, Stony Brook, New York, United States of America; 2 Department of Psychiatry and Behavioral Sciences, Feinberg School of Medicine, Northwestern University, Chicago, Illinois, United States of America; 3 Department of Psychiatry & Behavioral Neuroscience, University of Chicago, Chicago, Illinois, United States of America; University of Dundee, UNITED KINGDOM

## Abstract

The current study aimed to establish a fine-grained, efficient characterization of the concurrent neuropsychological contributions to social functioning in neuropsychologically-referred youth. A secondary aim was to demonstrate a useful statistic approach for such investigations (Partial Least Squares Regression; PLSR), which is underutilized in this field. Forty-five participants (70 – 164 months; M_age_ = 110.89; 34 male) were recruited from a large neuropsychological assessment clinic. Participants completed subtests from the NEPSY-II focusing on neuropsychological constructs that have been linked to social functioning (affect decoding, social memory, motor skills, visuomotor skills, response inhibition, attention and set-shifting, and verbal comprehension). Mothers completed the BASC-2, from which Atypicality and Social Skills scales were analyzed. PLSR revealed that difficulty with social memory, sensorimotor integration, and the ability to attend to and accurately discriminate auditory stimuli combine to best predict atypical or “odd” behavior. In terms of social skills, two factors emerged. The first factor indicated that, counterintuitively, greater emotional perception, visuospatial perception, ability to attend to and accurately discriminate auditory stimuli, and understand instructions was related to *poorer* social skills. The second factor indicated that a pattern of better facial memory, and sensorimotor ability (execution & integration) characterized a distinct profile of greater social ability. PLSR results were compared to traditional OLS and Backwards Stepwise regression approaches to demonstrate utility. Results also suggested that these findings were consistent across age, gender, and diagnostic group, indicating common neuropsychological substrates of social functioning in this sample of referred youth. Overall, this study provides the first characterization of optimized combinations of neuropsychological variables in predicting social functioning in assessment clinic-referred youth, and introduces to this literature a valuable statistical approach for obtaining such characterizations.

## Introduction

Challenges associated with social behavior are central concerns for many youth populations referred for neuropsychological testing [1–3]. Indeed, social challenges are often considered key, common, and treatment-refractory among such youth [4]. Traditionally, such challenges are rendered as either purely behavioral (i.e. reinforcement-driven) in nature [5, 6], arising from a lack of knowledge of correct social behaviors [7–12], or as complex, contextually-determined factors [13] that are difficult to characterize precisely. This view has largely influenced many interventions for social challenges, which have either focused on behavior modification [14–16], social contextual factors [14, 17, 18], or a bevy of putative, sometimes ill-defined target variables addressed at once [19, 20].

While the role of reinforcement, knowledge, and context are no doubt crucial to characterizing social deficits, increasing work suggests that lower-level, discrete, identifiable factors may play a key role in determining competent social interaction [21–24]. Under the broad rubric of “social performance-related factors” [20, 25, 26], such variables are thought to impede the effective reception, processing, and consequent output of social information [27–30]. Identification of these processes is important not only for uncovering how social behavior functions both within and across child clinical populations, but also for determining more precisely-identified targets for social intervention [26]. Crucially, such processes often map cleanly onto those commonly assessed in neuropsychological clinics. As such, examination of their role in social functioning in neuropsychologically-referred populations represents an ideal setting in which to consider their impact. While individual processes or populations have been explored in relation to social outcomes [15, 31–34], little work has sought to integrate multiple neuropsychological variables in determining social functioning outcomes. Moreover, given the interrelatedness of neuropsychological processes, a methodology is needed to determine the subtle interplay of factors that may be responsible for social functioning. We consider an array of well-specified individual neuropsychological variables in predicting concurrent social functioning in neuropsychologically-referred populations, and employ an under-used statistical approach to specify a configuration of these variables that optimally predicts social functioning.

### Common Neuropsychologically-Referred Populations with Social Challenges

Social deficits are often features of diagnoses such as autism spectrum disorders (ASD), attention-deficit/hyperactivity disorder (ADHD), learning and intellectual disorders (LD/ID), and anxiety/adjustment disorders. Deficits in social functioning are fundamental diagnostic characteristics of ASD and have been thoroughly documented in the literature [35–38]. Social functioning impairments are common in children diagnosed with ADHD, affecting 52%-82% of this population [39, 40]. Social deficits are increasingly seen as an important associated feature of this disorder, as this impairment has been reported by parents, teachers, and peers, and documented as early as preschool age [41]. Up to 75% of children with LD/ID also experience difficulties with social skills [42]. Finally, social deficits are common in children with anxiety/adjustment disorders as well; specifically, children who suffer from social anxiety are at an increased risk for experiencing low levels of social acceptance and more negative peer interactions [43, 44]. The range of severity and impairment level associated with these social deficits usually vary by age and gender for all disorders [45–47].

Interventions for social functioning often focus on improving social thinking; however, it may be that other perceptual and attentional processes interfere with social functioning in these populations irrespective of knowledge of social behavior [19, 28]. As previous studies have indicated [48–51], certain neuropsychological processes may act as potential mechanisms of social deficit in these youth. Thus, these mechanisms of social deficit may serve as targets in interventions for complex social behavior.

### Neuropsychological Predictors of Social Functioning

Recent research highlights several neuropsychological variables as especially promising in their contribution to social deficits in youth. First, the ability to accurately recognize and interpret nonverbal social cues (e.g., facial affect) is presumed to be important for effective social communication [52]. Children with ASD and ADHD demonstrate greater difficulty correctly identifying emotions than their typically-developing peers; this impairment contributes to the social skills deficits typically observed in these groups [53, 54]. Second, research has frequently demonstrated that individuals with characteristic social deficits (e.g., ASD) tend to have poor memory for social information [55, 56]. For instance, on a neuropsychological task assessing memory for visually-presented faces and objects, Hauck and colleagues (1988) found that children with ASD performed worse than their matched controls.

Third, the emergence of social competence in young children has been associated (chronologically and, some have argued, causally) with the development of motor skills [57, 58]. Thus, impaired motor development can deprive a child of the opportunity to play with other children which impacts their ability to simultaneously enhance motor and social skills [59]. Fourth, specific deficits in visuomotor integration skills have been increasingly linked to social skill impairment [24]. Fifth, deficits in response inhibition as well as attention and set-shifting have been linked to poorer social skills in children [22, 23]. These specific neuropsychological deficits are often characterized in children as frequent shifts in conversation, not listening to others, initiating conversations at inappropriate times, or frequently interrupting and intruding on others [23, 35]. These characteristics are most frequently observed in children with ADHD and co-occur with high rates of off-task, disruptive behavior, making them vulnerable to social rejection [23, 60]. Finally, verbal comprehension deficits have a clear impact on a child’s ability to adequately communicate with his or her peers. Individuals with social deficits often have marked language difficulties, including verbal comprehension, which may contribute to their social impairments [21].

However, little research has examined *concurrent* joint and relative contributions across multiple levels of neuropsychological analysis [61] to social deficits. Additionally, classical approaches to doing so tend to treat each predictor as wholly independent, which is very rarely the case in the context of neuropsychological assessment [62]. Thus, research is needed to more clearly elucidate the subtle interplay of neuropsychological factors that may contribute to social functioning in clinic-referred youth.

Moreover, analytic approaches that account for (or benefit from) highly intercorrelated predictors are needed to better ascertain the specific patterns of neuropsychological variables that may best predict concurrent social deficits. One such approach is Partial Least-Squares Regression (PLSR) [63, 64]. PLSR is a machine learning-type statistical technique that capitalizes on the use of many independent variables to optimize characterization of dependent variables. PLSR has several benefits that are highly applicable to this domain of modeling. First, it requires—and explicitly models—substantial covariation among predictors, facilitating estimation of interactions as well as redundancies. Second, it treats independent variables as *formative* (i.e. variables that contribute unique information that leads to a latent factor) rather than *reflective* (i.e. manifestations of a common underlying latent factor) [64, 65]. Formative indicators are free to correlate with one another (across the range of possible correlation), which supports more precise modeling of their interrelations. Third, PLSR includes in its modeling approach the internal cross-validation of effect estimates. That is, via one of several selection strategies (e.g. leave-one-out; random segments), the modeling procedure iteratively runs on a subset of the data, obtains estimates, and then applies these estimates to an independent subset of the data to obtain fit indices. Thus, indices of best fit in PLSR may be seen as a proxy for empirical generalizability of obtained independent variable patterns; as such, this approach addresses some of the usual questions applied to neuropsychology clinic-referred samples in terms of generalizability of effects. Overall, these features capitalize on structural features of neuropsychological data that have traditionally presented challenges to traditional regression models, and it is therefore important to introduce such approaches to this field. However, no analytic approach that benefits from such features has been used in this literature to date.

### Present Study

The current study first examined concurrent, multilevel neuropsychological contributions to social functioning. This was done using PLSR to capitalize on intercorrelation between independent variables and identify the unique combinations of variables that best predicted concurrent social functioning outcomes. This approach was compared to traditional multivariable regression models aiming to identify best predictors to demonstrate its utility. Given the theoretical and empirical differences in social functioning among children of varying diagnostic groups [30], ages [66], and gender [67], the differences in these combinations by each of these factors were also examined.

## Materials and Methods

### Participants

Forty-five participants (70–164 months; M_age_ = 110.89; 34 male) with primary diagnoses of autism spectrum disorders (ASD; 14.9%), Attention Deficit/Hyperactivity Disorder (ADHD; 34%), Learning Disorder/Intellectual Disorders (LD/ID; 40.4%), or Anxiety (2.1%) were drawn from a neuropsychological clinic-referred sample. They constitute the complete subset of a larger clinic-referred sample (see below) that completed the measures relevant to the present investigation.

### Procedures

Participants’ data used for this study was identified from among a sample of evaluations of children who were referred to the Pediatric Neuropsychology Service at the University of Chicago between 2002–2013 (N = 1500). Referrals for evaluation were typically received from pediatricians and treating specialty physicians to obtain a comprehensive assessment of the child’s neuropsychological status within the context of clinical concerns regarding functioning and learning. Children between the ages of two and emerging adulthood, with challenges affecting learning, behavioral regulation, emotional functioning, or social skills, comprise the broader clinic sample. All evaluations were conducted in the outpatient service and were directed and supervised by a licensed clinical psychologist with specialty training in pediatric neuropsychology. Tests selected for the assessment were based on clinical referral questions, and were administered and then scored by either a trained MA-level full-time psychometrician, or a graduate student trainee in clinical psychology who was certified as a psychometrician for the Service. Rechecking of scoring was undertaken for all cases. When warranted, formal clinical diagnoses were established utilizing DSM-IV-TR criteria. Families and referral sources were provided with a final report to guide intervention of academic, emotional/behavioral, and social needs.

At the first appointment, the child’s parent or legal guardian was presented with a written consent for their child’s participation in the clinical evaluation, and for their allowance of deidentified data from the assessment to be utilized in ongoing service research. Children utilized for this study were all from among those whose parents consented to both the assessment and data research use; consenting process and study procedures were approved by the University of Chicago Institutional Review Board. As noted above, given the nature of the referral, all children included in this study were presented with a clinical neuropsychological battery that included assessment of their intellectual functioning (see below), language expression and comprehension, verbal and visual memory, visuospatial processing and sensorimotor abilities, attention, executive functioning, and emotional and behavioral status. Relevant to the study, all children received the measures discussed below; this led to the reduction in available participants for the study, given tests administered and diagnoses of concern. That is, the sample included here represents 100% of those who completed all of the measures described below.

### Measures

Participants completed the Developmental Neuropsychological Assessment, Second Edition (NEPSY-II) [68] subscales focusing on affect decoding (Affect Recognition), social memory (Memory for Faces), motor skills (Fingertip Tapping), visuomotor skills (Imitating Hand Positions), response inhibition (Arrows), attention & set-shifting (Auditory Response Set), verbal comprehension (Comprehension of Instructions). This measure has been well-validated (including during the norming stage) in the diagnostic groups employed in the present study, and employs standard scores that adjust for norms by age [51, 68–70]. Participants also completed standard IQ measures from either the Wechsler [71–73] or Differential Ability Scales [74] batteries. Mothers completed the Behavior Assessment System for Children, Second Edition (BASC-2) [75], which is well-normed across the age ranges and diagnostic groups used here. The BASC-2 Atypicality and Social Skills scales are key indicators of populations with common social deficits (e.g., ADHD and ASD) [76, 77], were analyzed.

### Data Analytic Plan

We first conducted preliminary analyses to detect deviations from normality, and judged whether extreme skewness and/or kurtosis precluded executing parametric analyses. We then computed bivariate correlations between measures to assess presence of sufficient collinearity to support the use of (PLSR). That is, PLSR requires all or most independent variables to be substantially intercorrelated in order to effectively obtain reliable estimates of combinations among them [64]. This is similar to factor analysis in that bivariate correlations must be obtained prior to multivariate estimates. Given the potentially incommensurate nature of the scales by which the predictors and outcomes are calculated, both parametric (Pearson’s *r*) and nonparametric (Spearman’s *rho*) correlations were conducted. Next, as is frequently done when deriving PLSR models [78], all variables were converted to Z-scores to facilitate appropriate scaling of derived linear combinations in PLSR.

To test our first exploratory hypothesis, that linear combinations of NEPSY-II variables related to affective processing (Affect Recognition, Memory for Faces), motor planning and execution (Fingertip Tapping, Imitating Hand Positions), inhibition (Arrows, Auditory Response Set), and linguistic processing (Comprehension of Instructions) could be used to optimize cross-sectional prediction of BASC-2 mother-reported child Atypicality, we fit a corresponding PLSR model. We then evaluated the appropriate number of independent variables by examining the Root Mean Squared Error of Prediction (RMSEP) of each PLSR component, using Random Segment Cross-Validation on the entire sample, seeking the smallest value after the intercept. Next, we examined the loadings for each of the NEPSY-II variables to theoretically define each obtained component. Next, we examined the amount of variance in the cross-sectional predictor variables explained by the chosen component to ensure it accounted for a large proportion (qualification of variance estimates completed in accordance with Cohen’s recommendations) [79]. Next, we specified the corresponding Ordinary Least Squares (OLS) regression model with the specified number of components, as well as a Backwards Stepwise Regression model (i.e. the traditional approach to identifying significant concurrent predictors in this literature), and compared the amount of variance in Atypicality explained by these models to the amount explained by the PLSR model to ensure that predictive power was maximized. To test our second hypothesis, that discrete linear combinations could likewise be used to predict concurrentBASC-2 Social Skills, we repeated this process.

Finally, we conducted post-hoc analyses to examine whether the derived loading patterns were optimally effective for each of three subgroups (Learning Disorders, ADHD, Autism Spectrum Disorders) of participants. We did this by creating dummy coded variables for each subgroup. Then, we specified an OLS regression with three predictors: the subgroup indicator, each child’s score for the specified PLSR component, and the interaction between the two (if there was more than one PLSR component, this process was repeated for each component). If the interaction term was significant (*p* < .05), it was taken to indicate that the relation between the derived PLSR component and the BASC-2 outcome was especially descriptive of the given subgroup.

We note that the present sample size (*n* = 45) is sufficient to conduct PLSR [64]. Indeed PLSR has been effectively used with samples as small as 10 cases [65], and PLSR often produces greater statistical power than traditional multiple regression [63]. A past Monte Carlo simulation indicates that *N*~50 is sufficient to model at least 2–3 latent factors [65]. Additionally, PLSR statistical power is affected by normality of the distribution of observed variables [80]; thus, the Z-score transformation discussed above serves to increase statistical power with the present data.

## Results

### Descriptive Statistics

Participants were, on average, roughly 9.8 years old, mostly right-handed, and mostly male (see Table 1). They also exhibited IQ at the low end of the average range, and the majority received a primary diagnosis of a Learning Disability, Communication Disorder, or ADHD. While their performance was generally in the average range on most listed NEPSY-II subtests, average performances were just below the average range on Imitating Hand Positions and Arrows. They were roughly 1 standard deviation above the population mean for Atypicality and below the mean for Social Skills. Scores on the NEPSY-II did not differ by diagnostic group (in one-way ANOVA, all *p* > .07).

**Table 1:** Descriptive Statistics.

Variable	Mean (SD) [%]^3^
Age (months)	118.73 (25.13)
IQ	86.05 (16.17)^4^
Affect Recognition^1^	9.02 (3.17) [22.2]
Memory for Faces^1^	7.47 (3.36) [40.0]
Fingertip Tapping (Dominant)^1^	11.47 (4.00) [2.2]
Imitating Hand Positions^1^	6.80 (2.89) [51.1]
Arrows^1^	6.96 (3.49) [42.2]
Auditory Response Set^1^	7.73 (3.66) [35.6]
Comprehension of Instructions^1^	7.27 (3.14) [37.8]
Atypicality^2^	61.60 (15.32)
Social Skills^2^	42.00 (12.54)
	N (%)
Youth Gender	32 male (71.1%)
Handedness	34 right (75.6%)
Primary Diagnosis	Learning Disability or Communication Disorder = 19 (42.2%); ADHD = 16 (35.6%); Autism Spectrum Disorder = 5 (11.1%); Intellectual Disability = 1 (2.2%); Anxiety and/or Adjustment = 1 (2.2%); N/A = 3 (6.7%)

^1^NEPSY-II subtest scores represent scaled scores, with a normative mean of 10 and Standard Deviation of 3.

^2^BASC-2 subscale scores represent T-scores, with a normative mean of 50 and Standard Deviation of 10.

^3^Percent of the sample that is >1 Standard Deviation below the normative mean (i.e. NEPSY-II scaled score < 7).

^4^IQ scores represent standard scores, with a normative mean of 100 and Standard Deviation of 15 (IQ scores available for all but 2 participants).

### Bivariate Correlations

In terms of relations between NEPSY-II subtests and BASC-2 subscales, Pearson and Spearman correlations indicated that better Memory for Faces scores cross-sectionally predicted less Atypicality (see Table 2). However, better Comprehension of Instructions scores also predicted *poorer* concurrent Social Skills. BASC-2 Atypicality and Social Skills were negatively correlated.

**Table 2:** Bivariate Correlations Between Predictor and Outcome Variables.

Variable	1.	2.	3.	4.	5.	6.	7.	8.	9.
1. Affect Recognition	1	.32	.13	.24	.29	.49**	.34*	-.10	-.11
2. Memory for Faces	.36*	1	-.10	.48**	.31*	.32*	.24	-.32*	-.01
3. Fingertip Tapping (Dominant)	.23	.12	1	.07	.07	.14	.07	-.11	.15
4. Imitating Hand Positions	.23	.49**	.24	1	.29	.32*	.29	-.21	.08
5. Arrows	.32*	.30*	.21	.25	1	.44**	.33*	-.10	-.13
6. Auditory Response Set	.51***	.28	.25	.25	.46**	1	.47**	-.23	-.19
7. Comprehension of Instructions	.32*	.28	.11	.29	.27	.42**	1	-.01	-.40**
8. BASC-2 Atypicality	-.06	-.32*	-.04	-.25	-.06	-.22	-.04	1	-.36*
9. Social Skills	-.12	.04	.16	.14	-.13	-.20	-.43**	-.37*	1

Correlations above diagonal are Pearson product-moment correlations. Below diagonal are Spearman’s noparametric correlations. All significance tests are two-tailed.

*p < .05

**p < .01

***p < .001.

Among NEPSY-II subscales, Affect Recognition was correlated with Auditory Response set and Comprehension of Instructions (as well as Memory for Faces and Arrows performance only using Spearman correlations). Memory for Faces was correlated with Imitating Hand Positions and Arrows (as well as Auditory Response Set only using Pearson correlations). Imitating Hand Positions correlated with Auditory Response Set only using Pearson correlations. Arrows correlated with Auditory Response Set, as well as Comprehension of Instructions only using Pearson Correlations. Auditory Response Set correlated as indicated above, and also with Comprehension of Instructions. Fingertip Tapping did not correlate significantly with any other measure.

### PLSR Analyses

**Fig 1:**
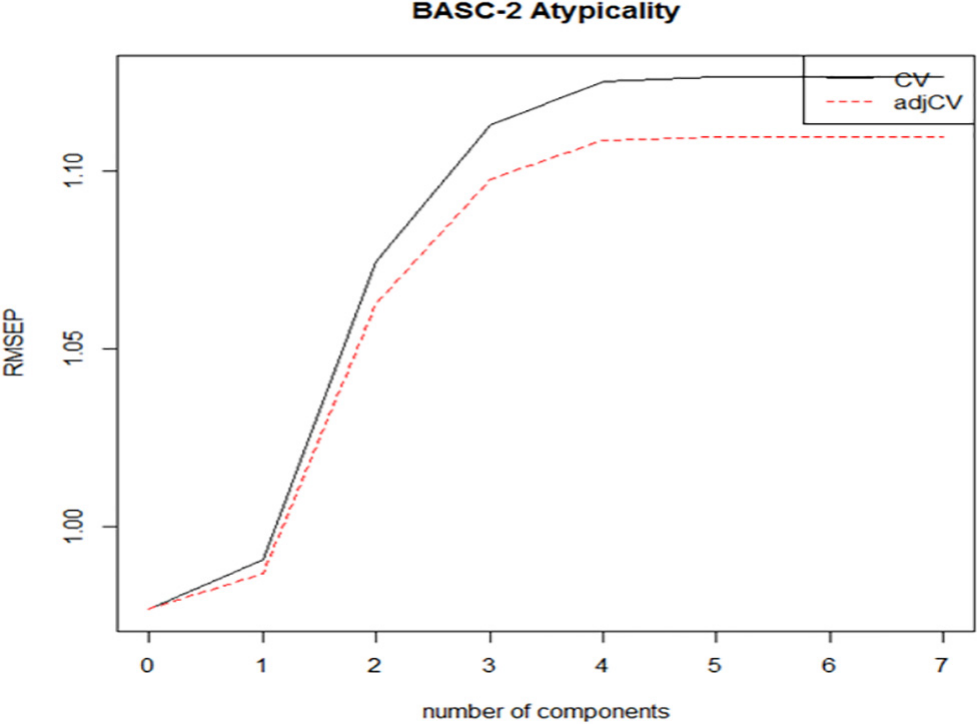
Root Mean Square Error of Prediction (RMSEP) values for determining the optimal number of Partial Least Squares Regression components for predicting concurrent BASC-2 Atypicality. CV = Cross-Validation.

In predicting greater concurrent Atypicality, one component composed primarily of poor performance on memory for faces, imitation of hand positions, and auditory attention & response set was optimal (see Table 3; Fig 1). This component accounted for a medium amount of the variance in Atypicality, and a large amount of the variance in the predictor variables.

**Table 3:** PLSR Factor Predicting BASC-2 Atypicality.

	Factor 1 Loadings
Affect Recognition	-.35
Memory for Faces	**-.54**
Fingertip Tapping	-.17
Imitating Hand Positions	**-.46**
Arrows	-.37
Auditory Response Set	**-.54**
Comprehension of Instructions	-.29
Cross-Validation RMSEP	.98
Adjusted Cross-Validation RMSEP	.98
*R* ^*2*^ for X variables	.361
*R* ^*2*^ for Y variable	.111

NEPSY-II subtest score loadings. RMSEP = Root Mean Square Error of Prediction. **Bold loadings** = ≥ .40.

**Fig 2:**
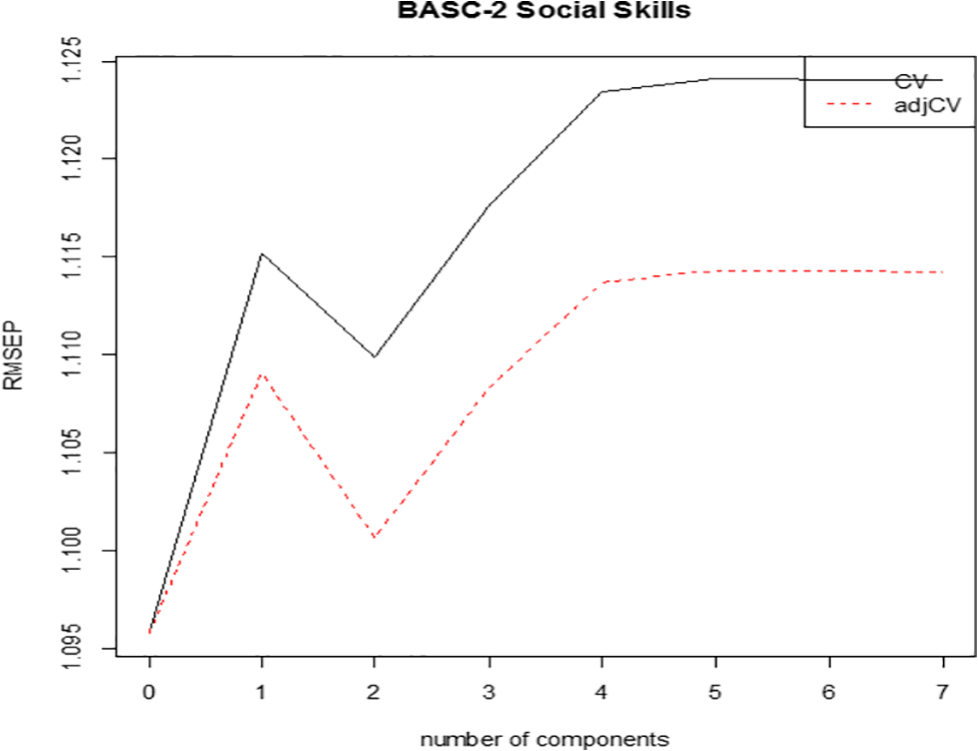
Root Mean Square Error of Prediction (RMSEP) values for determining the optimal number of Partial Least Squares Regression components for predicting concurrent BASC-2 Social Skills. CV = Cross-Validation.

For Social Skills, two components were optimal. The first component was composed of *poorer* affect recognition, arrows, auditory response set, and comprehension predicting *greater* concurrent Social Skills. This component accounted for a large amount of the variance in Social Skills and the independent predictor variables. The second component was composed of *greater* memory for faces, fingertip tapping, and imitation of hand positions predicting greater concurrent Social Skills (see Table 4; Fig 2). This component independently accounted for a small-medium amount of the variance in Social Skills and a large amount of the variance in the predictor variables.

**Table 4:** PLSR Factors Predicting Concurrent BASC-2 Social Skills.

	Factor 1 Loadings	Factor 2 Loadings
Affect Recognition	**-.40**	.28
Memory for Faces	-.31	**.50**
Fingertip Tapping	.18	**.40**
Imitating Hand Positions	-.20	**.59**
Arrows	**-.45**	.29
Auditory Response Set	**-.65**	.33
Comprehension of Instructions	**-.65**	
Cross-Validation RMSEP	1.115	1.110
Adjusted Cross-Validation RMSEP	1.109	1.101
*R* ^*2*^ for X variables	.284	.500
*R* ^*2*^ for Y variable	.168	.232

RMSEP = Root Mean Square Error of Prediction. **Bold loadings** = ≥ .40.

### Comparison to OLS and Backwards Stepwise Regression

For Atypicality, the initial OLS model (*F* = 1.03, *p* = .43) contained no significant predictors and had an *R* ^*2*^ of .16. The final Backwards Stepwise (*F* = 4.93, *p* = .032) model contained one predictor (memory for faces; *β* = -.32, *p* = .032) and had an *R* ^*2*^ of .10. Thus, the PLSR accounted for more variance than the Backwards Stepwise model while maximizing the use of available predictors.

For Social Skills, the initial OLS model (*F* = 1.65, *p* = .15) contained one significant predictor (comprehension of instructions; *β* = -.44, *p* = .013) and had an *R* ^*2*^ of .24. The final Backwards Stepwise (*F* = 8.25, *p* = .006) model contained one predictor (comprehension of instructions; *β* = -.40, *p* = .006) and had an *R* ^*2*^ of .16. Thus, the PLSR accounted for much more variance than the Backwards Stepwise model (indeed, it accounted for a comparable amount to the OLS model) while again maximizing the use of available predictors.

### Moderation by Diagnosis, Age, and Gender

With BASC-2 outcomes regressed on extracted factor scores, neither diagnostic status (all *p* > .39), age (all *p* > .27), or gender (all *p* > .75) moderated effects of derived factors on outcomes.

## Conclusions

This study had two aims. The first aim was to identify the combination of neuropsychological factors that best predict concurrent parent-reported social functioning in neuropsychologically-referred youth. The second aim was to showcase an underutilized analytic technique for doing so. These analyses reveal valuable information about basic neuropsychological processes subtending mother-reported atypicality and social skills in this population. Results suggest that challenges with social memory, sensorimotor integration, and the ability to attend to and accurately discriminate auditory stimuli combine to best cross-sectionally predict atypical or “odd” behavior. In terms of social skills, contrary to expectations, greater emotional perception, visuospatial perception, ability to attend to and accurately discriminate auditory stimuli, and understand instructions was related to *poorer* social skills. This suggests that basic capacity for these abilities may not translate into real-world functioning in these youth. Additionally, a pattern of better facial memory, and sensorimotor ability (execution & integration) characterized a distinct profile of greater social ability, consistent with recent basic research in ASD. Results also suggest that these findings are consistent across age, gender, and diagnostic group, indicating common neuropsychological substrates of social functioning in this sample of referred youth.

In terms of atypicality, these results indicate that imprecise or clumsy motor movements, and poorer ability to remember others’ faces and maintain and shift cognitive sets, are especially impactful in terms of being perceived as socially “odd”. These results are consistent with findings in both the ASD [76, 81, 82] and ADHD [77, 83] literatures, especially in their implication of visuomotor and executive functions. A child with this profile of difficulties may present with an inability to fluidly and quickly navigate spatial *and* social situations, yielding a uniformly atypical presentation. Future studies should examine more closely the degree to which this profile may be especially implicated in these disorders and, importantly, how intercorrelated they are within individuals (i.e. how frequently do face memory deficits co-occur with visuomotor deficits) across diagnostic groups to potentially provide insights into common neural mechanisms (e.g. cerebellum).

In terms of the first social skills factor, the finding that stronger affect recognition, response inhibition, ability to set shift, and capacity to comprehend instructions were related to *poorer* social skills was surprising, and is somewhat difficult to interpret. One possibility is range restriction; that is, this population exhibited poorer-than-average social skills, and so perhaps within an already-impaired group, these factors may lead children to engage more persistently (but not more skillfully) in social interaction. This finding converges with a small recent literature on ASD populations, indicating that especially socially motivated or persistent youth exhibit greater social deficits [84]. Conversely, this finding may stem from the nature of the social skills informants used in this study. Populations with social deficits tend to exhibit inflated ratings of their own social skills relative to parent report [85–87], with the size of this discrepancy sometimes relating to relative objective skill of the child. That is, children with slightly less significant deficits may show greater discrepancies from their parent. It is possible that these findings represent a mechanism by which this occurs, though studies specifically linking these processes to informant discrepancies are needed.

In terms of the second social skills factor, motor and visuomotor skills, as well as face memory, related to greater social skills. This finding converges well with usual findings in the literature indicating that skilled motor behavior [30, 58] and the ability to accurately recognize and engage with individuals [88] are valuable for competent social functioning. This factor is in some sense notable for what it does *not* include; that is, typical putative indices of social skills (e.g. emotion recognition and receptive language) did not appear to play a role. Future research should more clearly identify this phenotype, and explore whether the benefits of these variables (e.g. face memory), may be obtained in absence of theoretically closely-related ones (e.g. affect recognition).

This study also aimed to highlight the utility of the PLSR analytic technique in this literature. To our knowledge, PLSR has never been used for this purpose in the child neuropsychology literature. Our results reveal a number of advantages over OLS or Backwards Stepwise Regression models. Relative to of these models, the PLSR models demonstrated a more subtle and efficient use of the predictors; indeed, if one used these models, several variables that appear (in combination) to account for a considerable amount of variance in both outcomes would be discarded, leaving either one or zero remaining predictors in each model. Such approaches fail to capture the interplay and intercorrelation among these variables, and may spuriously suggest that neuropsychological indicators are only minimally related to the outcomes of interest. Compared to the Backward Stepwise regression models, PLSR also accounted for more variance in the outcome variables *while maintaining all predictors in the model*, achieving the aim of these models more efficiently. Compared to OLS models, the permitted intercorrelations facilitated identification of multiple predictors with limited loss of statistical power.

These findings are valuable for considering appropriate treatment options for social challenges in neuropsychologically-referred youth. Most notably, they suggest that specific, sometimes counter-intuitive underlying capacities may be targeted to yield effects on more distal, complex social behaviors. For instance, results suggest that training participants in face memory and sensorimotor skills may have valuable impact in reducing atypicality and increasing social skills (though direct training studies are needed to examine whether such causal relations indeed exist); conversely, training affect recognition (a common feature in social skills interventions) [4, 20, 30] may be less impactful on real-world outcomes of interest. Indeed, the first social skills factor suggests that training may be required in the opposite direction, though replication of such findings is certainly warranted before prescriptive suggestions are made.

It is important to note the limitations of this study. The sample was constrained in terms of the clinical profile and level of need (e.g., children specifically referred for a neuropsychological assessment), and consisted mostly of males with a somewhat limited range of diagnoses (e.g., mostly Learning Disability, Communication Disorder, and ADHD). Moreover, differences in established neuropsychological profiles of these populations may aggregate in fairly unique ways, which may contribute to some of the more counterintuitive results of the study; while the moderation analyses do not suggest these differences, this may likewise be the result of the small number of participants within each diagnostic group. This study also used very specific measures. While several subtests were utilized, outcomes were derived from one measure—the NEPSY-II. Moreover, while the selected subtests represent constructs that have been previously linked to social functioning (and reflect the preliminary nature of this investigation), other subtests and related variables (e.g. Theory of Mind) may play similar—or yet more important—roles. Additionally, only mothers completed the BASC-2, limiting the context of observation and attendant attributions for social behaviors [89]; future research should utilize multiple informants for each participant. Statistically, the Cross-Validation approach was able to provide some insight into the generalizability of the obtained linear variable combinations. However, in absence of a larger or more diverse sample (and without clearer thresholds for the RMSEP metric), the degree of generalizability to the broader population of neuropsychologically-referred participants (and, conversely, model overfitting) cannot be clearly specified. Relatedly, we specified PLSR models with the lowest RMSEP values *after* the intercept. Ideally, PLSR models are most instructive when the selected number of factors demonstrates an RMSEP that is *lower* than the intercept value; thus, while these models are helpful for illustration, they do not represent the strongest possible PLSR models in terms of their statistical properties.

Overall, findings highlight the importance of neuropsychological processes (and more sophisticated modeling techniques) as (exhibitors of) potential mechanisms of social deficit in these youth. As such, they serve as potential targets in interventions for complex social behavior. Future research should build on this study by continuing to investigate neuropsychological contributions to social skills deficits by utilizing a more comprehensive battery and multiple informants, along with appropriately-nuanced analytic techniques to further elucidate these important patterns of variable relationships.
